# Characterisation of Polyamines and Their Biosynthetic Pathways Contributing to Postharvest Anthracnose Resistance in Mango ( *Mangifera indica* L.)

**DOI:** 10.1111/pbi.70525

**Published:** 2026-01-04

**Authors:** Bei Zhang, Limei Huang, Qingbiao Xie, Hongli Luo, Qiannan Wang, Bang An

**Affiliations:** ^1^ State Key Laboratory of Tropical Crop Breeding, Tropical Crops Genetic Resources Institute Chinese Academy of Tropical Agricultural Sciences Haikou/Sanya Hainan Province People's Republic of China; ^2^ State Key Laboratory of Tropical Crop Breeding, School of Breeding and Multiplication (Sanya Institute of Breeding and Multiplication), School of Tropical Agriculture and Forestry (School of Agriculture and Rural Affairs & School of Rural Revitalization) Hainan University Sanya Hainan Province People's Republic of China; ^3^ Rubber Research Institute Chinese Academy of Tropical Agricultural Science Haikou Hainan Province People's Republic of China

**Keywords:** biosynthetic pathways, Jasmonic acid (JA) signalling, mango anthracnose, *p*‐coumaroyl‐feruloyl‐putrescine (CFP), plant defence, polyamines

Polyamines and their derivatives, such as phenolamides, play essential roles in plant defence systems, especially under biotic stress conditions (Gerlin et al. 2021). Anthracnose, caused by *Colletotrichum gloeosporioides*, is a major postharvest disease affecting mango (*Mangifera indica L*.), leading to significant economic losses during storage and transportation. Sustainable approaches that leverage mango's intrinsic defence mechanisms offer promising alternatives for managing postharvest diseases. This study identifies *p*‐coumaroyl‐feruloyl‐putrescine (CFP), a phenolamide derived from polyamines, as a critical metabolite in mango's defence against anthracnose, and investigates its biosynthesis, regulation, and antifungal efficacy.

To identify key metabolites involved in mango's response to anthracnose, we conducted a time‐course metabolomic analysis of infected (T1d to T3d, 1–3 days post‐inoculation) and control (CK0d to CK3d) fruit samples, with each sample comprising three biological replicates. Using UPLC–MS, we identified 175 distinct alkaloids, including phenolamides (Table S1). Metabolite values were normalised using *Z*‐scores before performing orthogonal partial least squares discriminant analysis (OPLS‐DA). The OPLS‐DA model revealed distinct metabolic profiles between infected and control samples, highlighting infection‐driven metabolic reprogramming (Figure 1a). Among the metabolites, CFP emerged as the most significant, exhibiting the highest score and a pronounced accumulation during the early stages of infection (Figure 1a–c; Tables S2 and S3). These findings suggest that CFP plays a central role as a rapidly induced defence metabolite during the initial response to anthracnose.

**FIGURE 1:**
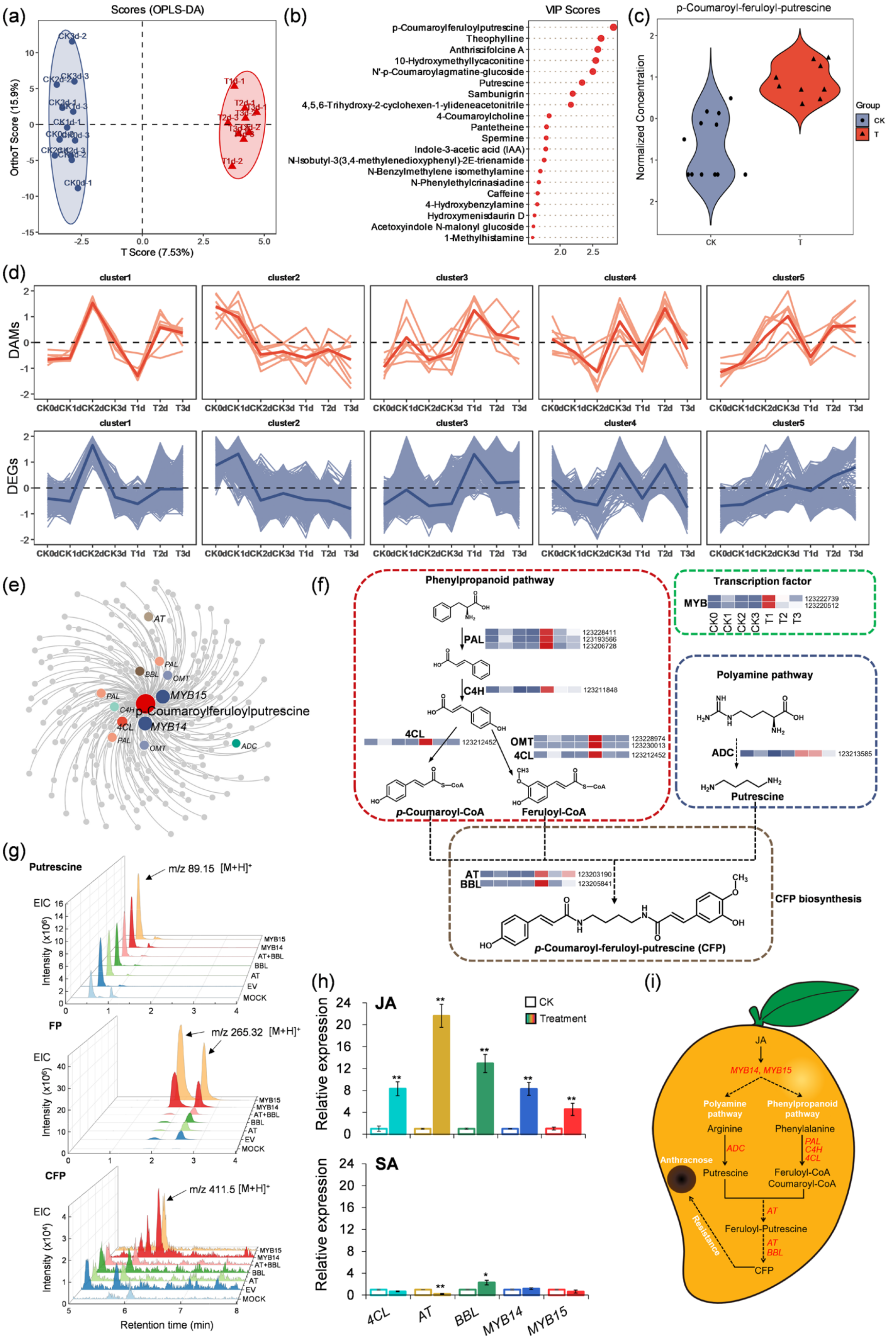
Metabolomic and transcriptomic analyses reveal the role of CFP in mango's defence against anthracnose. (a) OPLS‐DA of alkaloid profiles from control (CK0d–3d) and inoculated (T1d–3d) mango fruit samples at different time points (0–3 dpi). (b) Violin plot showing the normalised concentration of *p*‐Coumaroyl‐feruloyl‐putrescine (CFP in CK and T samples. (c) Variable Importance in Projection (VIP) scores for key metabolites contributing to the separation between CK and T groups. (d) *k*‐means clustering analysis of differentially accumulated metabolites (DAMs) and differentially expressed genes (DEGs) across control (CK) and treated (T) samples. (e) Co‐expression network identifying key genes associated with CFP biosynthesis. (f) Schematic of CFP biosynthesis pathways. Heatmaps indicate the expression levels of critical biosynthetic genes during infection. (g) LC–MS analysis of Putrescine, Feruloyl‐Putrescine (FP), and CFP in *Nicotiana benthamiana* leaves transiently expressing target genes (EV, empty vector; MOCK, untreated). (h) Expression profiles of CFP biosynthetic genes under exogenous Jasmonic acid (JA) and salicylic acid (SA) treatments. Bars represent the mean lesion diameter ± standard deviation. Asterisks represent statistically significant differences from the control: *p < 0.05; **p < 0.01. (i) A proposed model illustrating the biosynthetic pathway and regulatory network involved in the production of CFP in mango fruit in response to anthracnose infection.

To elucidate the biosynthetic pathways underlying CFP production, we conducted a transcriptomic analysis to identify genes associated with CFP production (Tables S4 and S5). *k*‐means clustering was performed using the Euclidean distance metric. Metabolites and genes were grouped into five distinct clusters (Figure 1d). Cluster 3, containing CFP and seven other metabolites, displayed a sharp increase at 1 and 2 dpi, followed by a decline at 3 dpi, reflecting an early and transient defence response. The genes in cluster 3 showed expression profiles closely mirroring the temporal patterns observed in metabolomic cluster 3, confirming a coordinated activation of metabolic and transcriptional defences. In this cluster, a series of genes involved in phenylpropanoid and polyamine pathways were identified. Co‐expression analysis further linked these genes to CFP biosynthesis (Figure 1e; Table S6), suggesting that phenylpropanoid and polyamine pathway genes are temporally and spatially regulated to regulate CFP biosynthesis.

Then we proposed the biosynthetic pathways leading to CFP synthesis by integrating both the phenylpropanoid and polyamine pathways (Figure 1f; Table S7). Enzymes in the phenylpropanoid pathway, including phenylalanine ammonia‐lyase (PAL), cinnamate‐4‐hydroxylase (C4H), 4‐coumarate‐CoA ligase (4CL), and caffeic acid O‐methyltransferase (OMT), were significantly upregulated during infection. These enzymes synthesise the acyl donors p‐coumaroyl‐CoA and feruloyl‐CoA (Wang et al. 2023). Similarly, arginine decarboxylase (ADC), a critical enzyme in the polyamine pathway, showed elevated expression, producing putrescine as the amine donor for CFP synthesis. The final steps of CFP biosynthesis involve the conjugation of p‐coumaroyl‐CoA and feruloyl‐CoA with putrescine, catalysed by acyltransferase (AT) and berberine bridge enzyme‐like (BBL) proteins (Bai et al. 2022).

To identify regulatory elements involved in CFP biosynthesis, we focused on MYB14 and MYB15, two Jasmonic acid (JA) responsive transcription factors (He et al. 2020). These regulators showed strong co‐expression with CFP biosynthetic genes (Figure 1e), indicating their central role in activating CFP production. Functional validation of CFP biosynthetic genes was performed using transient overexpression in *Nicotiana benthamiana* leaves (Figure 1g; Table S8). Overexpression of individual biosynthetic genes did not significantly increase CFP accumulation. However, the expression of MYB14 or MYB15 led to substantial production of CFP and its precursor, feruloyl‐putrescine (FP), reinforcing the regulatory role of these transcription factors in orchestrating the phenylpropanoid and polyamine pathways. Additionally, exogenous JA treatments significantly enhanced the expression of MYB14, MYB15, and CFP‐related genes, further supporting the role of JA as a key hormonal signal driving phenolamide‐mediated defence. In contrast, salicylic acid (SA) treatments had minimal effects on CFP biosynthetic gene expression (Figure 1h).

To assess the antifungal efficacy of CFP, its precursors, putrescine and FP, were selected due to the unavailability of commercially standardised CFP compounds. We conducted in vitro and in vivo assays (Figure S1). In vitro antifungal activity was evaluated by incorporating putrescine and FP into potato dextrose agar (PDA) medium and measured fungal colony growth to assess inhibitory effects. For in vivo assays, mango and apple fruits were inoculated with *C. gloeosporioides* conidia, followed by the application of putrescine or FP to the infection site. In vitro tests revealed that neither putrescine nor FP inhibited fungal growth on PDA plates, indicating limited direct antifungal activity under these conditions. However, in vivo assays demonstrated significant reductions in anthracnose lesion sizes on mango and apple fruits treated with putrescine or FP. FP exhibited greater protective effects than putrescine, reducing lesion sizes by approximately 30% compared to untreated controls. This context‐specific activity suggests that CFP and its precursors function by modulating host defences and fortifying structural barriers, rather than directly inhibiting fungal growth (Zeiss et al. 2021).

In conclusion, CFP plays a pivotal role in mango's defence against anthracnose, synthesised through the coordinated action of phenylpropanoid and polyamine pathways and regulated by MYB14 and MYB15 under JA signalling (Figure 1i).

## Author Contributions

B.A. and B.Z. designed the project. B.Z., L.H., Q.X., and Q.W. performed the experiments. B.Z., L.H. and Q.W. analysed the data. B.A., B.Z., Q.W. and H.L. wrote the manuscript.

## Funding

This research was funded by the Central Public‐interest Scientific Institution Basal Research Fund for CATAS (1630032022007); National Natural Science Foundation of China (32160594, 32260042).

## Supporting information


Figure S1.

Tables S1–S8.

## Data Availability

The data that support the findings of this study are available in the Supporting Information of this article.
