# Supplementary material for: Characterisation of Polyamines and Their Biosynthetic Pathways Contributing to Postharvest Anthracnose Resistance in Mango ( Mangifera indica L.)
Source: Plant Biotechnol J. 2026 Jan 4;24(5):2765–7. doi: 10.1111/pbi.70525 (PMC13110140; doi:10.1111/pbi.70525)
Supplement: Supplementary file 1 — Figure S1. [file PBI-24-2765-s001.docx]

**Supplementary Figures**


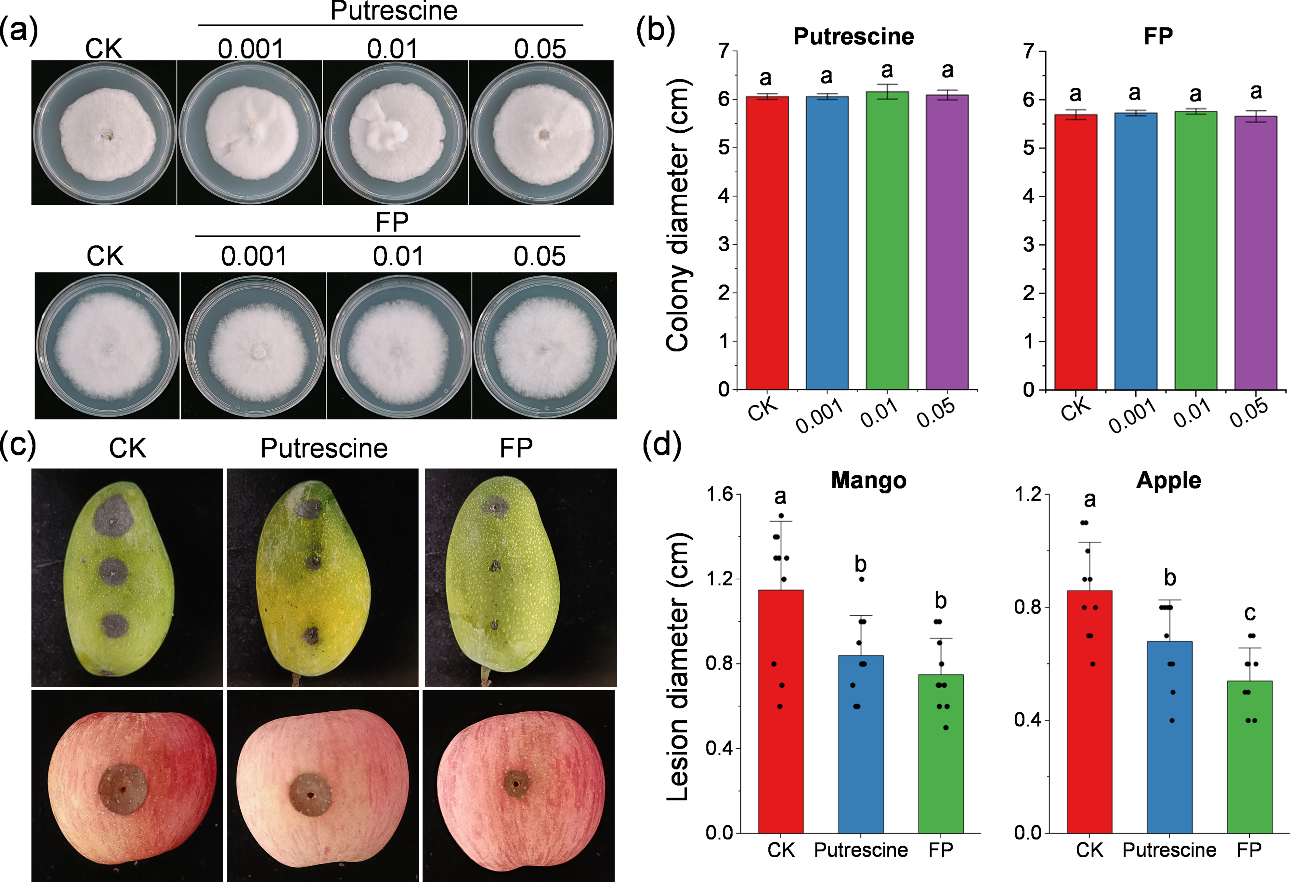


**Figure S1. Evaluation of anti-anthracnose activity of putrescine and feruloyl-putrescine (FP) *in vitro* and *in vivo*.** (a) *In vitro* colony growth of *C. gloeosporioides* on potato dextrose agar (PDA) medium supplemented with putrescine and FP at concentrations of 0.001, 0.01, and 0.05 mol L^-1^. (b) Colony diameter (cm) of *C. gloeosporioides* in response to putrescine and FP treatments. Bars represent the mean ± standard error for each treatment. (c) *In vivo* lesion development on mango and apple fruits treated with control (CK), putrescine, and FP, followed by inoculation with *C. gloeosporioides*. (d) Lesion diameters (cm) on mango and apple fruits. Bars represent the mean lesion diameter ± standard deviation for each treatment group. Different letters above the bars indicate statistically significant differences (p < 0.05).

**Supplementary Tables**

**Table S1** Identification of alkaloid metabolites in mango fruits During *Colletotrichum gloeosporioides* infection.

**Table S2** Variable importance in projection (VIP) scores of the identified metabolites in Mango fruits.

**Table S3** Differentially accumulated metabolites in mango fruits during *Colletotrichum gloeosporioides* infection*.*

**Table S4** RNA-seq analysis of gene expression in mango fruits during *Colletotrichum gloeosporioides* infection.

**Table S5** Differentially expressed genes in mango fruits during *Colletotrichum gloeosporioides* infection.

**Table S6** Correlation analysis between different accumulated metabolites and different expressed genes.

**Table S7** Genes highly correlated with CFP biosynthesis in Mango fruits.

**Table S8** Primers used in this study.
